# First person – Ahmed Mohamed

**DOI:** 10.1242/dmm.053027

**Published:** 2026-05-26

## Abstract

First Person is a series of interviews with the first authors of a selection of papers published in Disease Models & Mechanisms, helping researchers promote themselves alongside their papers. Ahmed Mohamed is first author on ‘ Dolutegravir developmental toxicity is mitigated by magnesium and folate in zebrafish embryos’, published in DMM. Ahmed is Research Assistant II in the lab of Baylor College of Medicine, Houston, TX, USA, investigating developmental biology and toxicology, especially how environmental chemicals and toxic exposures affect embryonic development, cell signalling and long-term health outcomes.

**Figure:**
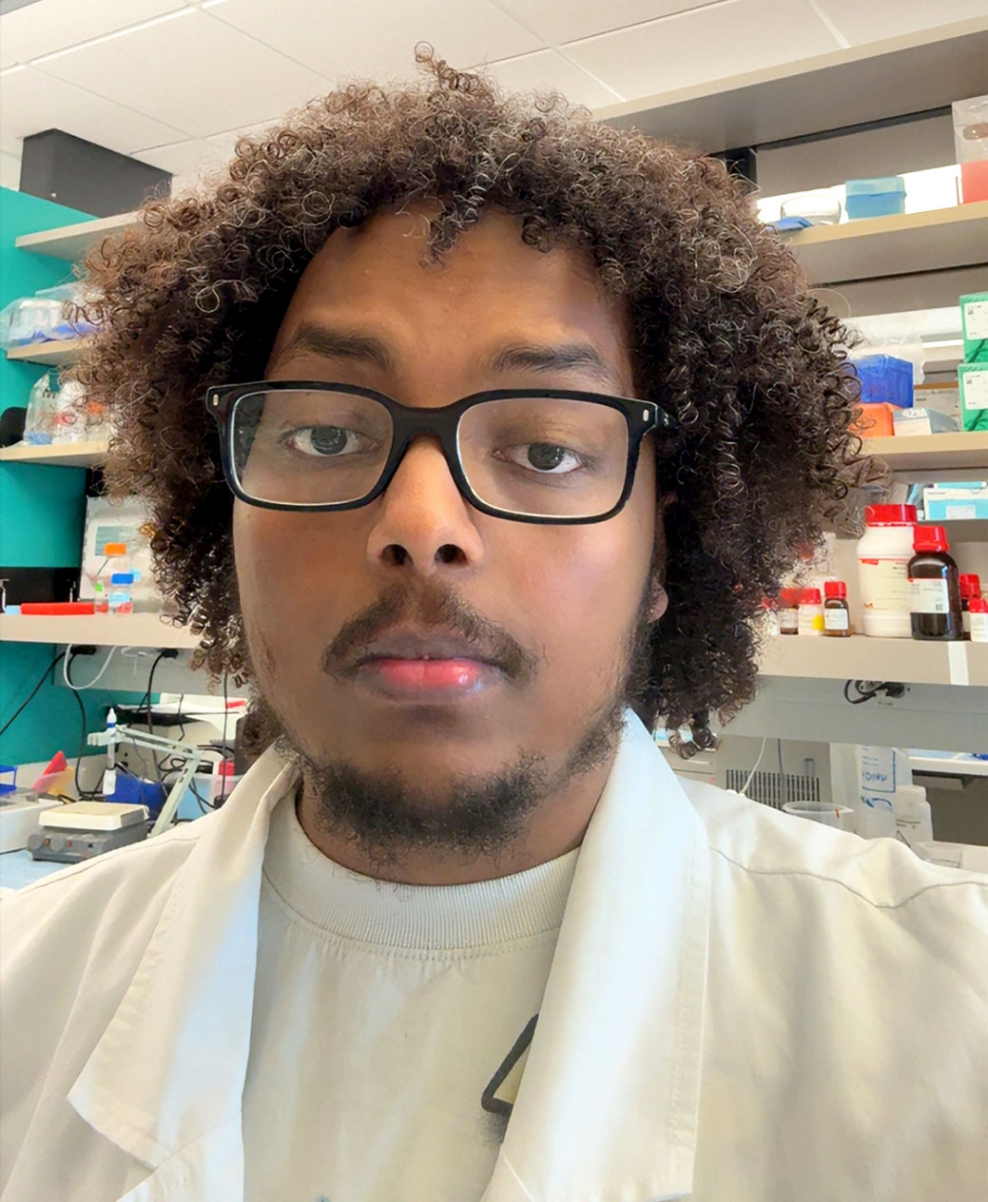
Ahmed Mohamed

**Who or what inspired you to become a scientist?**

What inspired me to become a scientist really came from a series of moments rather than just one thing. I remember in high school being genuinely fascinated watching chemical reactions happen in real time; it made me start asking why things work the way they do. Then, during my time in college, taking anatomy courses really deepened that curiosity. By the time I got to graduate school, what really solidified it for me was learning about so many diseases that still don't have cures. Also understanding the biochemical mechanisms by which these diseases function. That's when it shifted from just curiosity to purpose. I realized I didn't just want to understand science I wanted to be part of solving those problems. So, for me, it wasn't one single inspiration, but a progression from curiosity, to understanding, to wanting to make an impact.

**What is the main question or challenge in disease biology you are addressing in this paper? How did you go about investigating your question or challenge?**

In this study, we were trying to understand whether the HIV drug dolutegravir can cause developmental toxicity, and what molecular pathway is being impacted. We were also interested in exploring ways to reduce this toxicity. To do that, we used zebrafish embryos as our model system – both normal fish and *folr1* mutant lines that have disrupted folate transport. We exposed them to dolutegravir during early development and looked at survival and physical abnormalities. Then we tested whether adding folate or magnesium could rescue the defects and compared how each group responded.

… the developmental effects of [dolutegravir] are influenced not just by the drug itself, but also by the availability of key nutrients like folate and magnesium

**How would you explain the main findings of your paper to non-scientific family and friends?**

This study looked at whether the HIV drug dolutegravir can interfere with early development and what factors might reduce that risk. Using zebrafish embryos, we found that exposure to the drug can disrupt normal development early on. However, we also saw that adding certain nutrients – especially folate (a vitamin) and magnesium – helped the embryos develop normally. We also found that embryos with reduced ability to transport folate were more sensitive to the drug, but magnesium still offered some protection even in those cases. This suggested that magnesium may help through an additional protective mechanism beyond folate. Overall, the main takeaway is that the developmental effects of this drug are influenced not just by the drug itself, but also by the availability of key nutrients like folate and magnesium.

**What are the potential implications of these results for disease biology and the possible impact on patients?**

From a disease biology perspective, the study suggests that zebrafish can be a useful model for studying how integrase inhibitors may affect early development, and for testing potential ways to reduce those effects. It also highlights a possible interaction between drug exposure, folate pathways and magnesium availability that could be important for understanding mechanisms of developmental toxicity. From a patient impact standpoint, the results suggest that micronutrients – particularly folate and magnesium levels – may influence how the body responds to this medication during early pregnancy. This supports the importance of ensuring adequate folate intake and raises the importance that monitoring or maintaining magnesium levels could also be beneficial in clinical settings where this drug is used.

**Figure:**
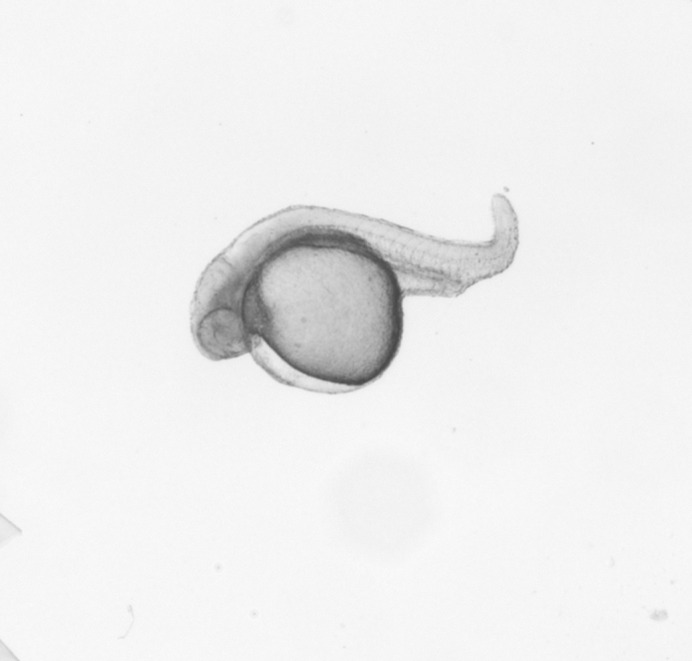
Representative image of an abnormal maternal-zygotic *folr1* mutant zebrafish embryo at 1 day post-fertilization.

**Why did you choose DMM for your paper?**

I chose Disease Models & Mechanisms because it is a well-respected journal that focuses on how diseases are studied using model systems. It also publishes a lot of work using zebrafish, which fits directly with the approach we used in our study.

**Given your current role, what challenges do you face and what changes could improve the professional lives of other scientists in this role?**

One of the biggest challenges in my current role is funding. Like many areas of biomedical research, progress is often limited by the availability and timing of grant support, which can make it difficult to plan long-term projects or expand on promising findings as quickly as we would like. A change that would improve the professional lives of scientists in this space would be more stable and sustained funding mechanisms, especially for early-career researchers. More consistent support would allow scientists to focus less on constantly securing funding and more on doing the actual research and driving discoveries forward.

**What's next for you?**

My next step is hopefully gaining acceptance into an MD/PhD program. My goal is to become a physician-scientist so I can both care for patients and continue doing research that directly improves outcomes for the population I study. I'm especially interested in translational research bridging what we discover in the lab with real clinical needs.

**Tell us something interesting about yourself that wouldn't be on your CV**

I previously ran a catering business focused on East African cuisine. It was a great experience that allowed me to showcase my culture to the city I call home.
